# Selenium Speciation and Stability in Selenium-Enriched Bean Sprouts During Cultivation and Processing

**DOI:** 10.3390/foods15081370

**Published:** 2026-04-15

**Authors:** Jiaqi Lu, Pan Yue, Xiting Huang, Jie Zheng, Shiyi Ou, Hua Zhou

**Affiliations:** 1 Department of Food Science and Engineering, Jinan University, Guangzhou 510632, China; 2 College of Chemistry and Materials Science, Jinan University, Guangzhou 510632, China; 3 School of Engineering, Guangzhou College of Technology and Business, Foshan 528138, China

**Keywords:** selenium speciation, bean sprouts, SeMet, thermal processing, enzyme activity, bioaccessibility

## Abstract

In selenium-rich foods, selenium speciation significantly impacts food quality. This study cultivated selenium-enriched bean sprouts using various selenium sources and systematically examined the dynamic changes in major selenium-containing compounds during thermal processing and storage. The results showed that selenomethionine (SeMet) is the predominant species in selenium-rich bean sprouts, and its stability is closely related to the redox enzymes in the bean sprout system. During processing or storage, SeMet is readily oxidized by reactive oxygen species into selenomethionine selenium oxides (SeMetO). However, SeMetO can be thermally reduced back to SeMet upon heating, revealing the reason for the stability of SeMet after processing. Additionally, the study elucidated the reduction kinetic equation of SeMetO, calculated its activation energy as 120.73 kJ·mol^−1^, and clarified the impact of coexisting proteins, cellulose, and other components on the reduction process. This research provides an important theoretical basis for the processing of SeMet in selenium-rich foods.

## 1. Introduction

Selenium (Se) is an essential trace element required for maintaining normal physiological functions in humans. Extensive evidence has demonstrated that selenium plays crucial roles in redox homeostasis, immune regulation, and cellular protection, primarily through its antioxidant, anti-inflammatory, chemopreventive, and antiviral activities [1,2,3,4,5]. These biological functions underscore the indispensable role of adequate selenium intake in preventing chronic diseases and sustaining immune competence.

Dietary intake constitutes the primary source of selenium for humans. However, selenium availability in foods is strongly influenced by soil selenium status. In China, previous nationwide surveys have indicated that approximately 51% of soils are selenium-deficient or contain low selenium levels, resulting in insufficient selenium accumulation along the food chain [6,7]. Consequently, it remains challenging for the general population to meet the recommended daily selenium intake for adults (approximately 30–40 μg day^−1^) through habitual diets alone [8]. Under these circumstances, the development of selenium-enriched foods has emerged as an effective and sustainable strategy to improve selenium nutritional status at the population level.

Among various selenium-biofortified foods, legume-based products have garnered particular attention due to their strong ability to assimilate inorganic selenium and convert it into bioavailable organic forms. Legume seeds can efficiently transform selenium into selenoamino acids, especially SeMet. In selenium-enriched legume products such as bean sprouts, selenium also predominantly exists in this form [9]. Selenium-enriched bean sprouts produced through controlled germination offer several advantages over animal-based or cereal-based selenium sources, including short production cycles, low costs, and simple processing requirements. Furthermore, studies have shown that selenium-enriched bean sprouts exhibit stronger antioxidant activity compared to conventional bean sprouts, further supporting their potential as functional foods [10]. Therefore, using bean sprouts as a dietary carrier for selenium biofortification represents a promising, economical, and easily implementable approach for selenium supplementation [11].

Owing to its structural similarity to methionine, SeMet can be non-specifically incorporated into plant proteins, serving as the major storage form of selenium in plant tissues [12]. Compared with inorganic selenium species such as selenite, which generally exhibit lower bioaccessibility, or methylselenocysteine (SeMC), which is chemically more labile, SeMet demonstrates higher chemical stability and is efficiently absorbed in humans via methionine transport pathways, resulting in superior bioaccessibility [13,14]. These characteristics make SeMet a key determinant of the nutritional quality of selenium-enriched plant foods.

Notably, the predominant selenium form in selenium-rich foods, particularly in selenium-enriched bean sprouts, significantly impacts food quality. The selenium content and speciation in selenium-enriched foods are highly susceptible to degradation during food processing and storage. Food processing represents a critical stage that can significantly alter both total selenium levels and selenium speciation. For example, substantial selenium losses have been observed during the withering and rolling processes of selenium-enriched tea, with selenium-methylselenocysteine exhibiting the most pronounced depletion, whereas SeMet shows relatively higher retention [15]. Similarly, extrusion processing has been reported to cause up to a 43.05% reduction in selenium content in selenium-enriched rice, while conventional steaming and microwave treatments result in minimal selenium loss [16]. Thermal processing such as boiling can further induce the leaching of organic selenium species, including SeMet, from plant tissues, leading to both quantitative losses and alterations in selenium speciation profiles [17].

In addition to processing, storage conditions pose another major challenge to selenium stability in plant-based foods. Although low-temperature storage can partially mitigate selenium degradation, its effectiveness depends on both storage temperature and selenium chemical form. Previous studies have shown that even under frozen storage (−24 °C), selenium losses ranging from 4.34% to 12.24% can occur in vegetables and algae within one month, highlighting the intrinsic instability of selenium species in agricultural products [18].

To date, research on selenium-enriched bean sprouts has primarily focused on cultivation strategies and selenium biofortification efficiency. In contrast, systematic investigations into the dynamic transformation of selenium species during their processing and storage remain very limited. Particularly critical is the fact that the key enzymatic activities, food matrix interactions, and redox-driven conversion pathways governing selenium species stability have not yet been comprehensively elucidated. Furthermore, existing conclusions regarding the stability and bioaccessibility of SeMet are largely based on studies under ideal conditions or in simplified systems. Its actual persistence and effectiveness within complex food matrices subjected to real-world processing chains still require in-depth investigation.

Therefore, the present study aimed to systematically investigate the transformation patterns and mechanisms of selenium species in selenium-enriched bean sprouts under typical processing and storage conditions. Specifically, we examined the roles of endogenous enzyme systems, food matrix components, and the redox environment in regulating the stability of SeMet, the predominant organic selenium species. By precisely characterizing these changes, this work seeks to provide a theoretical foundation for developing targeted processing and preservation strategies that maximize the retention, stability, and nutritional quality of selenium in selenium-enriched sprout-based foods, thereby enhancing their value in the functional food sector.

## 2. Materials and Methods

### 2.1. Materials and Chemicals

Mung bean seeds were obtained from Guangzhou, China, and germinated in our laboratory to produce mung bean sprouts. In the absence of a specific national standard for selenium-enriched sprouts, a selenium content of ≥75 μg kg^−1^ (dry weight basis) was adopted as the classification criterion, in accordance with the threshold for “selenium-rich” food claims defined in the Chinese National Food Safety Standard GB 28050–2011 [19].

All chemicals were of analytical grade or higher unless otherwise stated. Glucose oxidase (100.0 U mg^−1^), catalase (200.0 U mg^−1^), and oxidase activity assay kits were purchased from Shanghai Bide Pharmaceutical Technology Co., Ltd. (Shanghai, China). LC–MS–grade methanol, citric acid (≥99%), nitric acid, and ammonium hydroxide were obtained from Aladdin Reagent Co., Ltd. (Shanghai, China). Certified reference standards of sodium selenite, selenocystine (SeCys_2_), Se-methylselenocysteine (SeMC), and SeMet were supplied by the National Institute of Metrology (Beijing, China). Ultrapure water (18.2 MΩ·cm) was produced using a Milli-Q water purification system (Millipore, Billerica, MA, USA).

### 2.2. Cultivation of Selenium-Enriched Mung Bean Sprouts and Processing Simulation

Uniform mung bean seeds (80.0 g per batch) were selected and soaked in the dark for 5 h at 25 ± 1 °C in aqueous solutions containing 10 mg L^−1^ of different selenium sources: sodium selenite (Se(IV)), SeMet, or selenocystine (SeCys_2_). The soaking solution volume was maintained at a ratio of 3:1 (mL g^−1^) relative to dry seed weight. After soaking, the seeds were thoroughly rinsed with deionized water and transferred to a hydroponic germination system. Sprouts were cultivated in darkness for 5 days. A control group was prepared using tap water without selenium supplementation. Germination rate, sprout length, and fresh weight were recorded at the end of the cultivation period.

To simulate thermal processing, 1.0 g of fresh mung bean sprouts was mixed with 10.0 mL of deionized water in sealed tubes and heated in a temperature-controlled oil bath at 100 °C for 1, 3, 5, or 7 min. Immediately after heating, samples were rapidly cooled in an ice–water bath to terminate thermal reactions.

A selenium concentration of 10 mg L^−1^ was selected, based on the method described by Wang et al. [20], whose study focused on optimizing selenium sources for organic accumulation. For storage experiments, fresh sprouts were stored under refrigerated (4 °C) and ambient conditions. Prior to selenium analysis, samples were lyophilized, and dry weight was recorded. All analytical results are expressed on a dry weight basis unless otherwise stated.

### 2.3. Determination of Selenium Content and Speciation

Free selenium species were determined following references [21,22] with minor modifications. Briefly, 1.0 g of fresh sample was mixed with 10.0 g of ultrapure water in a 25.0 mL glass tube (sample-to-water ratio of 1:10, m/m). The mixture was boiled for 10 min to extract selenium species, followed by centrifugation at 8000 r/min for 15 min. The supernatant was collected for further analysis.

The HPLC-ICP-MS method for selenium speciation was adapted from Tie et al. [23] with optimized extraction conditions according to our laboratory setup. In that study, the spike recoveries for SeMet ranged from 89.5% to 103.2%, with RSD values below 8.5%, demonstrating good accuracy and reproducibility of the method in mung bean sprout matrix.

Under optimized conditions, the limits of detection (LOD) and quantification (LOQ) were as follows: for SeMet, LOD and LOQ were 220 μg kg^−1^ and 742 μg kg^−1^ (dry weight), respectively. For other selenium species (Se(IV), SeCys_2_, SeMC), the LOD and LOQ ranged from 140 to 223 μg kg^−1^ and from 465 to 742 μg kg^−1^ (dry weight), respectively.

See Appendix A for standard measurements and calibration curves.

### 2.4. Enzyme Activity Assays in Mung Bean Sprouts

Enzyme activities were assayed using three biological replicates, each measured in triplicate. For each assay, a blank control (reaction mixture without enzyme extract) and a heat-inactivated enzyme control (enzyme extract boiled for 10 min) were included to correct for non-enzymatic reactions.

For fresh bean sprouts, the dry weight of a 1.0 g sample taken from the root–stem junction was 0.0722 g (dry weight ratio: 7.22%). For sprouts stored at room temperature (open containers, ambient conditions), moisture evaporated progressively over time, increasing the dry weight ratio of the stored material. Specifically, 1.0 g of the room-temperature-stored sprouts collected on days 1, 2, and 3 yielded dry weights of 0.138 g, 0.33 g, and 0.81 g, respectively, reflecting cumulative moisture loss in the stored samples rather than in freshly harvested material. To normalize these samples to the same dry weight basis as fresh sprouts (0.0722 g), the fresh weights taken for analysis were adjusted to 0.523 g, 0.219 g, and 0.089 g on days 1, 2, and 3, respectively, with three replicates per time point. Refrigerated sprouts exhibited minimal moisture loss; to achieve the same 0.0722 g dry weight, 0.963 g, 0.914 g, and 0.870 g of fresh weight were used on days 1, 2, and 3, respectively, also with three replicates per time point. All samples for enzyme activity and selenium speciation analysis were thus normalized to the dry weight of fresh bean sprouts (0.0722 g), ensuring a consistent dry matter basis for comparison.

Samples subjected to different processing conditions were homogenized in an ice–water bath with the addition of 1.0 mL of 50 mM phosphate buffer (at the corresponding pH), added in two increments. The homogenate was centrifuged at 8000× *g* for 15 min at 4 °C. The supernatant was collected as the crude enzyme extract and kept on ice for immediate analysis.

#### 2.4.1. Polyphenol Oxidase (PPO) Activity

PPO activity was determined based on the enzymatic oxidation of catechol to *o*-benzoquinone. Briefly, 180 μL of catechol reaction solution (pH 6.0) was added to a microcuvette, followed by 20 μL of enzyme extract. Heat-inactivated enzyme extract served as the control. The increase in absorbance at 420 nm was monitored at 1 min intervals for 10 min at room temperature. One unit (U) of PPO activity was defined as an absorbance increase of 0.01 per minute.

#### 2.4.2. Catalase (CAT) Activity

CAT activity was determined based on its ability to decompose hydrogen peroxide. Residual H_2_O_2_ reacted with ammonium molybdate under acidic conditions to form a yellow complex. The reaction system contained 180 μL of ammonium molybdate reaction solution (pH 7.0) and 20 μL of enzyme extract. After incubation at 25 °C for 10 min, absorbance was measured at 405 nm. One unit (U) of CAT activity was defined as the amount of enzyme decomposing 1.0 μmol of H_2_O_2_ per minute per gram of sample.

### 2.5. Oxidation and Thermal Reduction of SeMet

SeMet was completely dissolved in deionized water and stoichiometrically oxidized using equimolar hydrogen peroxide to obtain SeMet oxide (SeMetO). Both SeMet and SeMetO solutions exhibited no detectable degradation during extended storage under ambient conditions.

Preliminary experiments indicated that thermal treatment could induce the reduction of SeMetO back to SeMet. To systematically investigate this transformation, the following experiments were conducted.

#### 2.5.1. Thermal Reduction Kinetics of SeMetO

Aliquots (2.0 mL) of SeMetO solution (5.0 mg L^−1^) were sealed in high-temperature -resistant stainless-steel tubes and heated under various temperature–time conditions: 60, 70, 80, 90 and 100 °C for 0.3, 2.0, and 5.0 h. Each condition was tested in triplicate. Reduction kinetics were analyzed by linear regression of ln(C_0_/Ct) versus heating time to obtain the reaction rate constant (*k*). Activation energy was calculated using the Arrhenius equation. The conversion of SeMetO to SeMet was monitored by LC–MS.

#### 2.5.2. LC–MS Analysis of SeMet and SeMetO

SeMet (5.0 mg) was dissolved in 2.0 mL deionized water, followed by the addition of equimolar hydrogen peroxide to achieve complete oxidation. The solution was gently vortexed and immediately analyzed by LC–MS. SeMetO samples heated at 100 °C for 10 min, 120 min, and 5 h were also collected and analyzed to monitor the thermal reduction process.

#### 2.5.3. NMR Characterization of SeMet and SeMetO

For NMR analysis, SeMet (50.0 mg) was dissolved in deuterated water (D_2_O), and ^1^H, ^13^C, and ^77^Se NMR spectra were recorded. After the addition of equimolar hydrogen peroxide, spectra were immediately reacquired. The oxidized sample was then heated for 15 min and 120 min, followed by final NMR measurements to track structural changes during thermal treatment.

### 2.6. Effects of Oxidative Enzymes on SeMet Stability

Oxidative enzymes activated during food processing or storage can generate reactive oxygen species (ROS), which may oxidize SeMet to SeMetO or promote further selenium degradation. To elucidate the influence of enzymatic oxidation on SeMet stability, glucose oxidase and catalase were employed as model oxidative enzymes.

#### 2.6.1. Oxidation by Glucose Oxidase

SeMet (5.0 mg) and glucose (8.8 mg) were dissolved in 1.0 mL deionized water. Glucose oxidase (8.8 mg) was added, and the reaction was carried out in an open container for 3.0 h at room temperature. Samples were collected at regular intervals for analysis of SeMet and SeMetO concentrations.

#### 2.6.2. Protection of SeMet by Catalase

SeMet (5.0 mg) was dissolved in 1.0 mL deionized water, followed by the addition of catalase (5.0, 10.0, or 15.0 mg). Hydrogen peroxide (10.0 μL) was then added, and the mixture was stirred for 30 min. Aliquots were collected during the reaction for analysis.

#### 2.6.3. Combined Enzymatic Effects

Two sequential enzymatic protocols were used to investigate combined effects:

Protocol A: Catalase followed by glucose oxidase. SeMet (5.0 mg) was dissolved in 1.0 mL deionized water, catalase (4.4 mg) was added, followed by glucose oxidase (8.8 mg). The reaction proceeded for 30 min with continuous stirring, followed by an additional 120 min with periodic sampling.

Protocol B: Glucose oxidase followed by catalase. SeMet (5.0 mg) was dissolved in 1.0 mL deionized water, glucose oxidase (8.8 mg) was added, and after 30 min, catalase (4.4 mg) was introduced. The reaction continued for 120 min with sampling at regular intervals.

### 2.7. In Vitro Bioaccessibility of Selenium

#### 2.7.1. Simulated Gastric Digestion

Fresh mung bean sprouts (0.50 g, *n* = 3) were placed in 50 mL centrifuge tubes, and 10.0 mL of simulated gastric fluid (10 g L^−1^ pepsin, 0.15 mol L^−1^ NaCl, pH 2.0) was added. Samples were incubated in a shaking water bath at 37 °C for 6 h. After digestion, samples were centrifuged at 3500× *g*, and the supernatants were filtered through 0.22 μm membranes and stored at 4 °C prior to analysis.

#### 2.7.2. Simulated Intestinal Digestion

Following gastric digestion, the pH was adjusted to 7.5 ± 0.2 using saturated NaHCO_3_ solution. Subsequently, 10.0 mL of simulated intestinal fluid (30 g L^−1^ pancreatin, 15 g L^−1^ amylase, 10 g L^−1^ bile salts, 0.15 mol L^−1^ NaCl) was added. The mixture was incubated at 37 °C for 6 h. After digestion, samples were centrifuged and filtered as described above.

#### 2.7.3. Calculation of in Vitro Bioaccessibility

In vitro bioaccessibility of selenium in the form of SeMet was calculated as:

In vitro bioaccessibility (%) = (SeMet content in digested supernatant/SeMet content in undigested sample) × 100

### 2.8. Effects of Food Components on SeMetO Reduction

Soy protein, pectin, and cellulose were selected as representative components of mung bean sprouts to evaluate their effects on the stability and reduction kinetics of SeMetO. The effects of component addition timing (before or after SeMet oxidation), addition level (4.0 or 10.0 mg), and thermal treatment conditions (70, 90, and 100 °C for 20 min, 2, 3, and 4 h) were systematically investigated. The reduction of SeMetO under these conditions was monitored by LC–MS.

### 2.9. Statistical Analysis

All experiments were conducted with three independent biological replicates. Data are presented as the mean ± SD. Statistical analyses were performed using SPSS software (version 26.0). The specific methods applied were as follows: in Section 3.1 (cultivation parameters), one-way ANOVA was used to assess the effects of selenium source, soaking time, or temperature on germination rate and sprout length; in Section 3.2 (thermal processing) and Section 3.3 (storage), two-way ANOVA was employed to analyze the main effects and interactions of treatment conditions with time or temperature on selenium species content and enzyme activity; in Section 3.2.3 and Section 3.3.3 (correlation analysis), Pearson correlation analysis was used. For ANOVA, Duncan’s multiple range test was applied for post hoc comparisons. Statistically significant differences (*p* < 0.05) are indicated by different lowercase letters (a, b) in the figures, while different uppercase letters (A, B) denote higher significance (*p* < 0.01).

## 3. Results and Discussion

### 3.1. Effects of Cultivation Parameters on Mung Bean Sprout Growth

**Figure 1:**
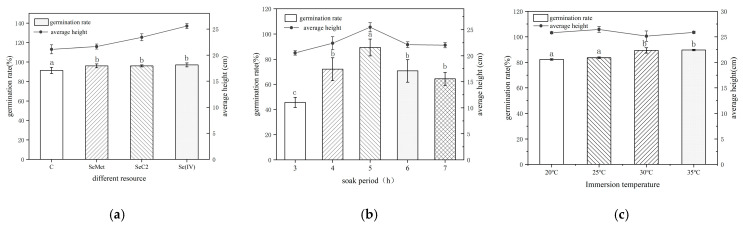
Growth and germination responses to key cultivation variables. (**a**) Effect of different selenium sources; (**b**) Effect of soaking duration; (**c**) Effect of soaking temperature. Different letters above bars indicate significant differences (*p* < 0.05, Duncan’s test).

Cultivation parameters are crucial for mung bean sprout growth and selenium enrichment. This study systematically evaluated three key factors at a fixed selenium concentration of 10 mg L^−1^ (Figure 1). The choice of selenium source significantly affected post-germination growth but not the initial germination rate. Hypocotyl elongation followed the order: selenite [Se(IV)] > selenocystine (SeCys_2_) > SeMet. The Se(IV) treatment yielded the greatest sprout length (26.80 cm), significantly exceeding the control (20.65 cm), followed by SeCys_2_ (24.69 cm) and SeMet (22.42 cm) (Figure 1a). In contrast, soaking time was a decisive factor for germination success (*p* < 0.05), exhibiting a biphasic response with a peak germination rate of ~87% at 5 h (Figure 1b), beyond which germination declined and decay increased. Soaking temperature within the 20–35 °C range had a minimal effect, causing less than a 10% variation in germination rate and no significant impact on final sprout height (Figure 1c). Consequently, the optimal cultivation parameters, aimed at maximizing both germination efficiency and hypocotyl growth, were determined to be: soaking seeds in 10 mg L^−1^ sodium selenite [Se(IV)] for 5 h.

The superior growth promotion by Se(IV) is likely attributed to its efficient uptake via phosphate transporters [24] and its role in modulating physiological processes such as antioxidant activity [25]. The established optimal conditions integrate findings from previous studies which indicated dose- and form-dependent effects, such as a 5 h soak in 30 μg mL^−1^ Na_2_SeO_3_ [26] or the use of 10 μmol L^−1^ Na_2_SeO_3_ [27], while also considering the potential adverse effects of higher doses.

These findings indicate that mung bean germination and early seedling development are governed by the coordinated action of multiple factors, with soaking time primarily regulating germination efficiency and selenium source type exerting a more pronounced influence on post-germination growth. This optimized cultivation framework provides a rational basis for subsequent investigations into selenium accumulation, speciation transformation, and stability during processing and storage.

### 3.2. Effects of Thermal Processing

#### 3.2.1. Influence of Thermal Processing on Selenium Speciation

In this study, mung bean sprouts were cultivated with three different selenium sources—sodium selenite, SeMet, and selenocystine—and their selenium composition was analyzed prior to thermal processing. The analysis revealed that SeMet constituted the highest proportion of organic selenium, while the level of SeMC was relatively low, a finding consistent with previous literature reports [28].

**Figure 2:**
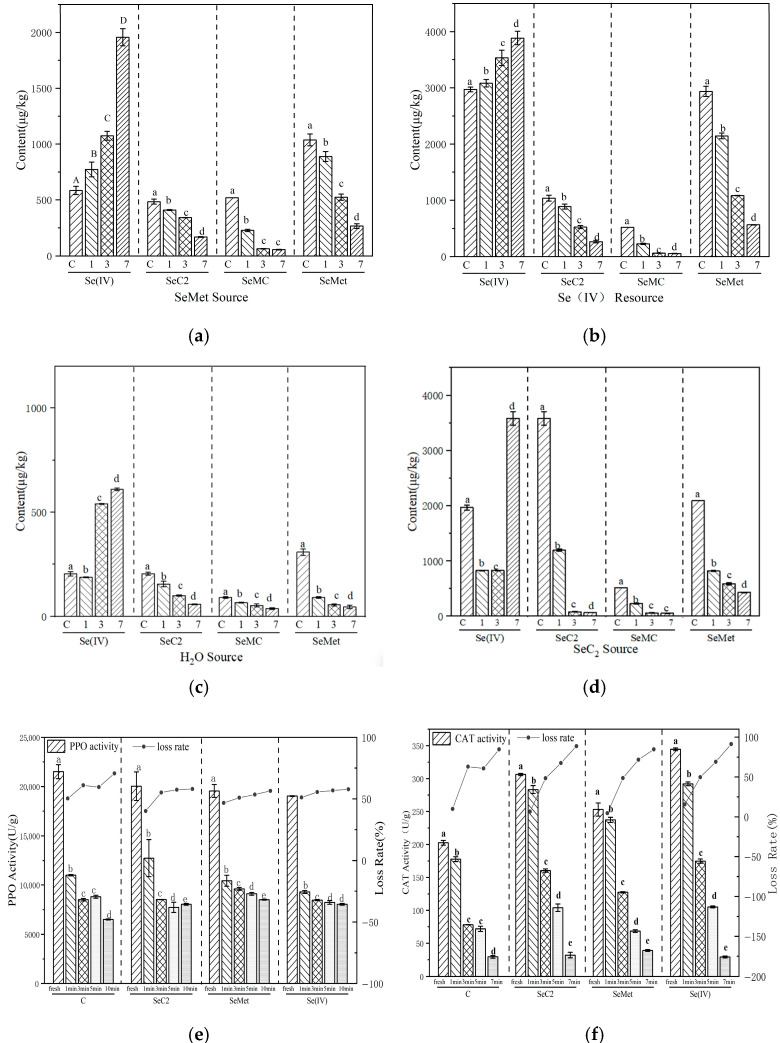
Changes in selenium speciation and enzyme activities in bean sprouts during heating. (**a**–**d**) Selenium speciation; (**e**,**f**) PPO and CAT activities. Significant differences are indicated by different letters (lowercase, *p* < 0.05; uppercase, *p* < 0.01).

Thermal processing induced significant changes in the selenium species distribution within the sprouts. A key observation was the marked increase in inorganic selenium [Se(IV)] content with prolonged heating time (Figure 2a–d). This increase was most pronounced in sprouts initially biofortified with Se(IV), where the content rose from 2985 to 3857 μg kg^−1^ (Figure 2b).

Concurrently, a general decrease in the content of all measured organic selenium species was observed; however, their thermal stability varied considerably. SeCys_2_ and SeMC were the most labile, whereas SeMet demonstrated the highest retention, indicating superior thermal stability. For instance, SeMC sustained a loss of approximately 80% after only 3 min of heating (Figure 2a), while the degradation of SeMet was significantly less pronounced.

This differential stability aligns with reported data highlighting the relative robustness of SeMet during heating [29]. The phenomenon is likely attributable to the higher bond dissociation energy of the C–Se bond in SeMet’s structure compared to the diselenide (Se–Se) bond present in SeCys_2_ [30].

Collectively, these results indicate that thermal processing shifts the selenium profile in sprouts toward a higher proportion of inorganic selenium. Given that SeMet is the predominant and highly bioavailable organic species, its relative stability is crucial for preserving the nutritional value of the processed product.

#### 3.2.2. Effect of Thermal Processing on Enzyme Activities

The shift in selenium speciation during thermal processing may be associated with changes in the activity of endogenous oxidative enzymes. To assess concurrent changes in the redox environment, the activities of PPO and catalase CAT were measured under the same heating conditions.

Thermal processing significantly altered the activities of two key oxidative enzymes—polyphenol oxidase (PPO) and catalase (CAT)—in mung bean sprouts (Figure 2e,f). These enzyme activities decreased with prolonged heating, but their thermal stability differed substantially. CAT activity decreased gradually in a time-dependent manner, whereas PPO demonstrated the highest thermal resistance. This variation is primarily due to differences in protein structure; PPO possesses a binuclear copper center within a tightly packed tertiary conformation, rendering it more resistant to denaturation [31].

The distinct inactivation kinetics of these enzymes likely modify the redox environment in the sprout matrix. Given that oxidative stress levels influence the stability of selenium-containing amino acids in foods, the differential loss of enzyme activities may be a contributing factor to the varying retention of organic selenium species—particularly the relatively heat-stable SeMet. To explore this relationship, we conducted Pearson correlation analysis between residual enzyme activities and the retention rates of major selenium species.

#### 3.2.3. Conclusion of Correlation Analysis in Thermal Processing

Pearson correlation analysis, based on data from three biological replicates (*n* = 3 per time point), demonstrated significant positive correlations between the residual activities of catalase (CAT) and polyphenol oxidase (PPO) and the retention of SeMet after heat treatment (r = 0.975 and 0.884, respectively; *p* < 0.01) (Table 1). This strong association suggests that the preservation of antioxidant enzyme activities may be linked to the enhanced stability of SeMet, potentially by helping to mitigate oxidative stress within the tissue. As will be shown in Section 3.4.3, model-system experiments provide direct mechanistic support for this interaction. They demonstrate that enzymatically generated H_2_O_2_ (via glucose oxidase) can oxidize SeMet, while the presence of catalase, by scavenging H_2_O_2_, effectively protects SeMet from oxidation. This evidence directly confirms that the redox activities of specific enzymes are capable of regulating the oxidation state of SeMet, offering a plausible chemical basis for the observed correlation between residual enzyme activity and SeMet retention in the complex sprout matrix.

Further analysis revealed interconnected changes in selenium speciation and enzyme activity profiles during processing. Heat treatment inactivated antioxidant enzymes such as CAT and PPO, which is expected to impair reactive oxygen species (ROS) scavenging and could contribute to an oxidative environment. This shift aligns with the observed oxidative degradation of the more labile organic selenium species (e.g., SeCys_2_ and SeMC). Concurrently, a decrease in SeMet was accompanied by an increase in inorganic selenium [Se(IV)]. One plausible explanation for this inverse trend, consistent with established selenium chemistry under stress, could involve the oxidative degradation of organic species, potentially via pathways such as molecular cleavage [32]. It is important to note that the specific conversion pathway from SeMet to Se(IV) was not directly traced in this study and remains a subject for further mechanistic investigation.

Taken together, these findings demonstrate that thermal processing coincides with a net shift in selenium speciation from organic to inorganic forms. This process appears closely associated with the disruption of the enzymatic antioxidant system and the resultant change in the redox landscape of the sprout. Within this context, SeMet—owing to its inherent chemical stability and as evidenced by its strong positive correlation with residual enzyme activity—emerges as the most retained organic species, thereby playing a determining role in the final nutritional selenium quality of processed, selenium-enriched sprouts.

### 3.3. Influence of Storage Conditions

#### 3.3.1. Effect of Storage on Selenium Speciation

**Figure 3:**
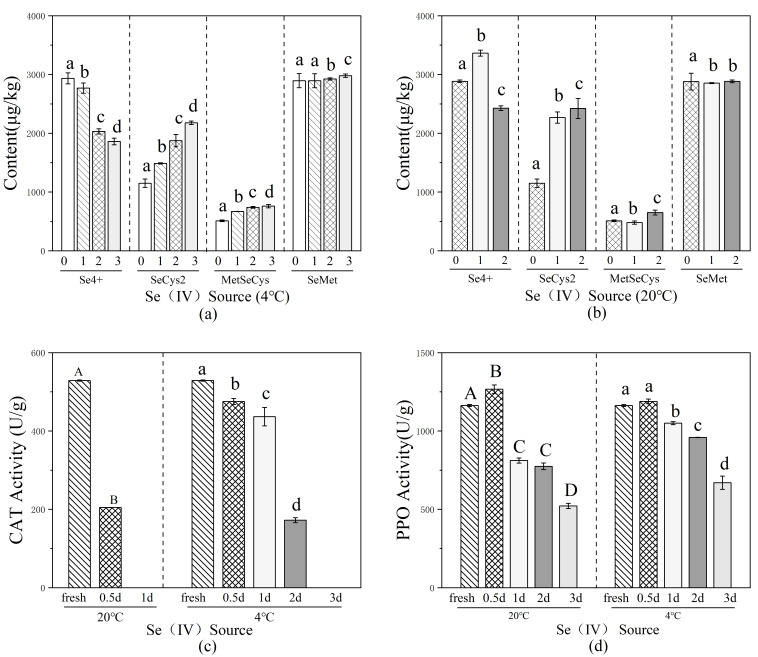
Changes in selenium speciation and enzyme activities in bean sprouts during storage at 4 °C and 20 °C. (**a**,**b**) selenium speciation; (**c**,**d**) activities of PPO and CAT in different storage. Different letters (a–d for *p* < 0.05; A–D for *p* < 0.01) denote significant differences (Duncan’s test).

Storage significantly altered selenium speciation in mung bean sprouts (Figure 3). The inorganic selenium [Se(IV)] content decreased continuously, with a more pronounced reduction under refrigeration (~37.0%) than at room temperature (~19.9%) in sprouts initially treated with Se(IV) (Figure 3a,b). Conversely, the three organic selenium species (SeCys_2_, SeMC, and SeMet) increased steadily. For example, SeMet content rose by approximately 7.75% in the Se(IV)-treated group (Figure 3a).

The decrease in inorganic Se(IV) alongside the increase in organic SeMet during storage could be attributed to (i) the ongoing, albeit slow, post-harvest metabolic assimilation of selenium [33], and/or (ii) the release of protein-bound selenium (e.g., from degraded selenoproteins) into the detectable free amino acid pool. The observed negative correlation between antioxidant enzyme decline and organic Se increase may reflect a parallel, but not necessarily causal, deterioration of cellular redox homeostasis and activation of proteolysis/metabolic changes, rather than oxidative stress directly driving Se(IV) assimilation.

#### 3.3.2. Dynamic Changes in Enzyme Activities During Storage

**Figure A3:**
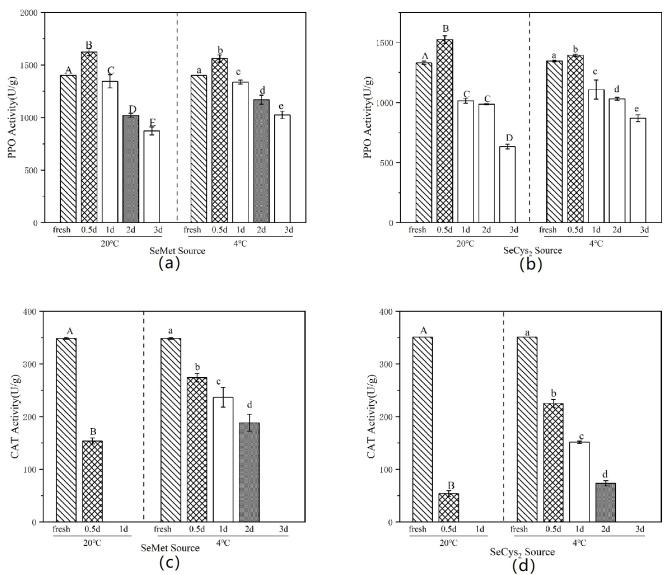
Changes in polyphenol oxidase (PPO) and catalase (CAT) activities in bean sprouts during storage at 4 °C and 20 °C. Sprouts were cultivated with different selenium sources: (**a**,**c**) SeMet-fortified group; (**b**,**d**) SeCys_2_-fortified group. Storage temperatures: left panels of each figure represent 20 °C; right panels represent 4 °C.Data with different letters are significantly different according to Duncan’s test (lowercase, *p* < 0.05; uppercase, *p* < 0.01).

The activities of oxidative enzymes changed markedly during storage (Figure A3). Both CAT and PPO activities exhibited a continuous decline. In terms of stability under ambient conditions, the enzymes could be ranked as PPO > CAT. This rapid loss indicates a compromised primary antioxidant defense system against hydrogen peroxide and a progressive shift in the internal oxidative-enzymatic landscape of the tissue.

Similar enzyme activity changes have been observed in selenium-enriched mushrooms during cold storage, where CAT and GSH-Px activities were initially upregulated to delay senescence before gradually declining [34].

#### 3.3.3. Conclusion of Correlation Analysis in Storage

During room temperature storage (Table 2), the activities of CAT and PPO were both significantly negatively correlated with the contents of SeCys_2_ and SeMet (e.g., r = −0.994 for PPO vs. SeCys_2_, *p* < 0.01), while no significant correlation was found between enzyme activities and inorganic Se(IV) content. Under refrigerated storage (Table 3), the pattern became more complex. The two enzymes showed highly significant negative correlations with organic selenium species (SeCys_2_ and SeMC), while they turned positively correlated with inorganic Se(IV) content (e.g., r = 0.939 for CAT vs. Se(IV), *p* < 0.01).

The consistent and condition-dependent correlations between enzyme activities and selenium speciation suggest that the enzymatic redox status is a key factor influencing selenium transformation during storage. The progressive decline in antioxidant enzymes (e.g., CAT, PPO) likely contributes to a shift in the intracellular redox state. This altered redox landscape may, in turn, create conditions that favor the observed redistribution of selenium species.

Notably, the relationship between declining enzyme activity and selenium speciation differs fundamentally between thermal processing and storage. During thermal processing, the rapid inactivation of oxidative enzymes through direct denaturation, coupled with the potential initiation of non-enzymatic oxidation reactions, may accelerate the degradation of organic selenium. In contrast, the gradual decline in enzyme activity during storage may occur alongside other bioconversion processes (such as microbial activity or slow selenium species migration), which could instead favor the accumulation or manifestation of organic selenium. This mechanistic divergence explains why a similar trend of decreasing enzyme activity can lead to opposite trends in organic selenium levels (e.g., SeMet) in the two processes.

Consequently, the stable, oxidation-resistant SeMet accumulates as the dominant organic form during storage, which may serve as a bioavailable selenium reservoir. The enzyme-activity correlations reported here help to elucidate potential internal drivers of the dynamic selenium redistribution observed during storage and provide a basis for future mechanistic investigations. It is important to note that this study did not directly measure the activities of specific selenium assimilation enzymes or key metabolic intermediates, and thus the exact mechanisms require further experimental validation.

From a food processing perspective, these findings suggest that for monitoring and controlling the activity of endogenous enzymes like CAT, PPO could be a novel strategy for preserving bioavailable selenium in plant-based foods. Techniques such as mild blanching to selectively inactivate oxidases, or the use of natural antioxidants, could be explored to maintain a favorable redox state during processing.

### 3.4. Oxidation and Thermal Reduction Behaviour of SeMet

To elucidate the intrinsic chemical Behaviour of SeMet under thermal and oxidative conditions, and to better interpret its apparent loss and transformation during heating and storage of selenium-enriched bean sprouts, a simplified model system was established. In this system, the oxidation of SeMet and the subsequent thermal reduction of its oxidized product were systematically investigated.

#### 3.4.1. LC-MS and NMR Characterization of SeMetO and Its Thermal Reduction

##### Preparation and Structural Confirmation of SeMetO

In solution, SeMet was quantitatively oxidized using an equimolar amount of hydrogen peroxide to produce SeMetO. The conversion process and product structure were confirmed by liquid chromatography-mass spectrometry (LC-MS) and nuclear magnetic resonance (NMR) spectroscopy.

**Figure 4:**
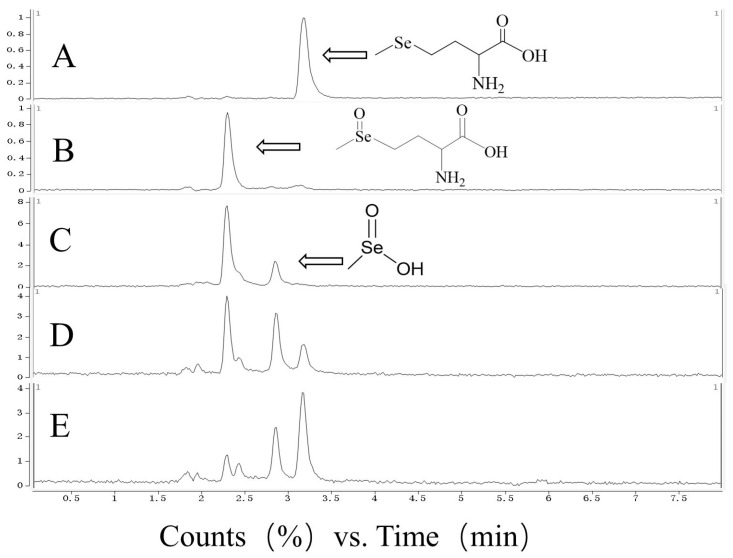
HPLC-MS analysis of the oxidation and reduction process of SeMet after treatment with H_2_O_2_ and heating at 90 °C. (**A**) Starting material SeMet; (**B**) Oxidation product SeMetO; (**C**) SeMetO heated at 90 °C for 10 min; (**D**) SeMetO heated at 90 °C for 30 min; (**E**) SeMetO heated at 90 °C for 60 min.

LC-MS monitoring showed that SeMet was completely converted to SeMetO in less than 1 min (Figure 4), as indicated by the shift of the major peak retention time from 3.166 min to 2.290 min, demonstrating that the oxidation was rapid and complete.

**Figure 5:**
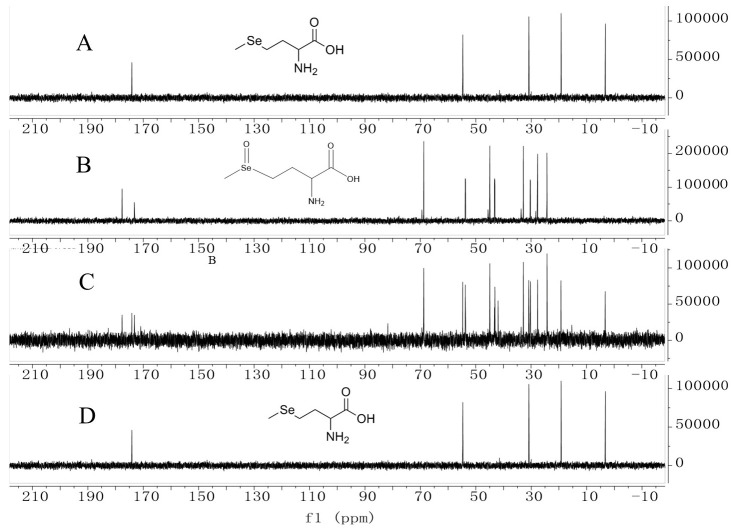
NMR-monitored reduction of SeMetO ((**A**): carbon NMR spectrum of SeMet; (**B**): spectrum after oxidation with equimolar H_2_O_2_ to form SeMetO; (**C**): spectrum of SeMetO heated for 15 min; (**D**): spectrum of SeMetO heated for 120 min).

Further NMR (Figure 5) analysis provided direct evidence for the structural transformation: the characteristic Se–CH_3_ proton signal of SeMet at approximately 1.9 ppm completely disappeared, and a new signal attributable to Se(O)CH_3_ emerged at about 2.4 ppm. The ^13^C NMR spectrum displayed paired resonances for each carbon signal, consistent with the formation of two diastereomeric selenoxide species. Together, these data confirm the successful preparation and structural integrity of SeMetO.

##### Thermal Reduction Behaviour of SeMetO

The thermal stability and reduction Behaviour of SeMetO were investigated under controlled heating conditions. LC-MS tracking analysis indicated that when heated at 90 °C, SeMetO was progressively and completely converted back to the starting material, SeMet, with extended heating time.

To clarify the conversion pathway, a time-resolved ^13^C NMR analysis of the heating process was performed. After heating the SeMetO solution at 100 °C for 15 min, a new signal appeared at 19.21 ppm in the spectrum, assigned to the Se–CH_3_ carbon of the reduction product SeMet. Simultaneously, characteristic Cβ signals of SeMet appeared around 31 ppm. Correspondingly, the intensity of the SeMetO-specific Cβ signal at approximately 69 ppm decreased significantly. Upon continued heating for 120 min, the signals associated with SeMet further intensified, while those of SeMetO were substantially diminished. These consistent spectral changes clearly demonstrate the thermally induced cleavage of the Se=O bond and the complete reduction of SeMetO back to SeMet.

#### 3.4.2. Kinetic Characteristics of SeMetO Thermal Reduction

**Figure 6:**
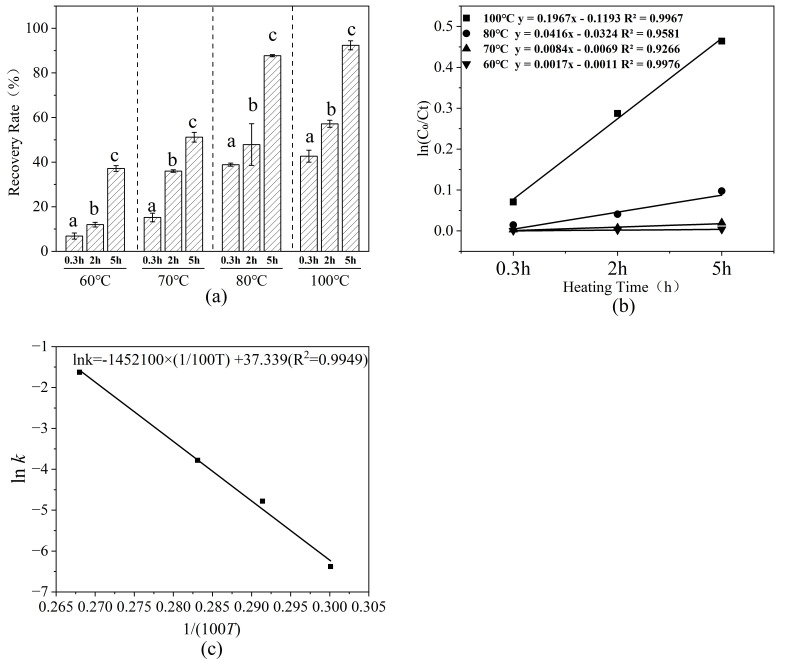
Effect of simulated thermal processing on the reduction of SeMetO. (**a**) Reduction rate of SeMetO at different heating temperatures; (**b**) Kinetic curves of thermal reduction at different temperatures; (**c**) Arrhenius plot of the fitted first-order rate constant, depicting lnk as a function of the reciprocal temperature 1/*T* (where *T* is temperature in *K*). The linear fit yields a coefficient of determination R^2 ^= 0.9949. Data with different letters are significantly different according to Duncan’s test (*p* < 0.05).

To quantitatively describe the thermal reduction behaviour of SeMetO, kinetic analyses were conducted at different temperatures and heating durations. As shown in Figure 6a, the reduction rate increased markedly with temperature under static heating conditions, and complete conversion of SeMetO was achieved after 5 h at 100 °C.

The experimental data were well fitted by a first-order kinetic model. Plots of ln(C_0_/Ct) versus time showed good linearity at all tested temperatures, with R^2^ values ranging from 0.9266 to 0.9976 (Figure 6b). Based on the Arrhenius plot (Figure 6c), the activation energy (Ea) was 120.73 kJ·mol^−1^ and the pre-exponential factor (A) was 1.74 × 10^16^. The relatively high activation energy indicates that the reduction of SeMetO is highly sensitive to temperature, explaining why this reaction proceeds slowly under mild conditions but becomes dominant during high-temperature processing. These kinetic parameters provide a mechanistic basis for understanding the apparent “loss–recovery” behaviour of SeMet observed during thermal treatment of selenium-enriched foods.

#### 3.4.3. Enzyme-Mediated Modulation of SeMet Stability

##### Effect of Glucose Oxidase on SeMet Oxidation

**Figure 7:**
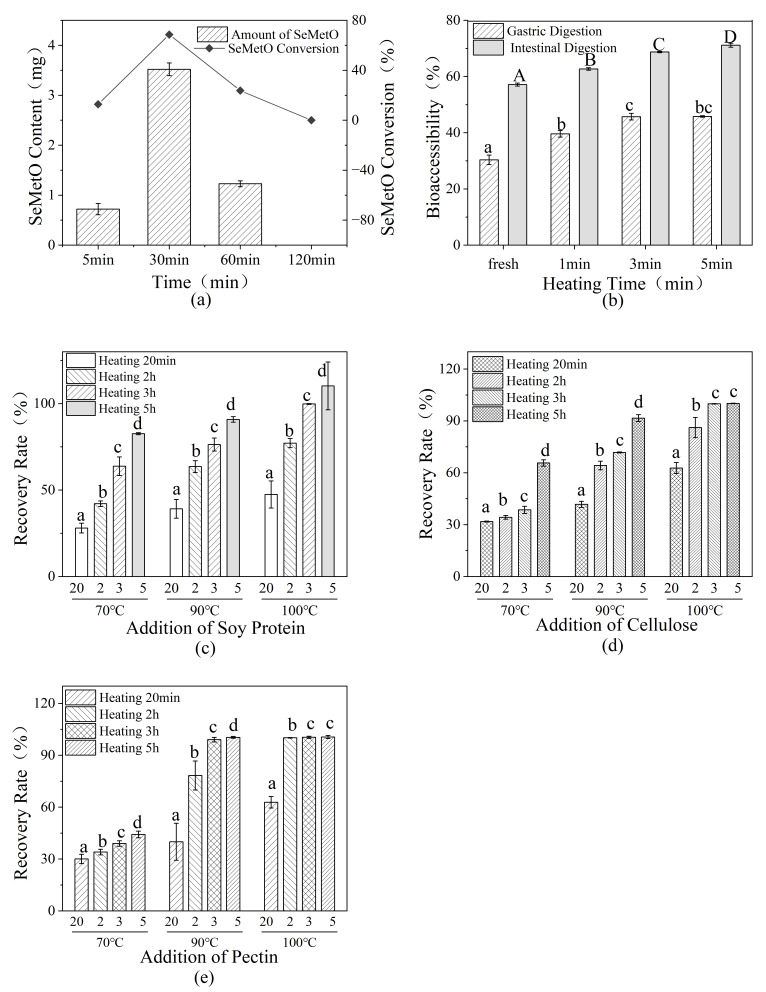
Multifactorial modulation of SeMet stability and SeMetO reduction. (**a**) Oxidase effect on SeMet; (**b**) thermal effect on SeMet bioaccessibility; (**c**) effect of soy protein; (**d**) effect of cellulose; (**e**) effect of pectin. Data with different letters are significantly different according to Duncan’s test (lowercase, *p* < 0.05; uppercase, *p* < 0.01).

To simulate enzymatically induced oxidative stress, SeMet was incubated in a glucose oxidase system with glucose as the substrate. Under these conditions, SeMetO formation occurred rapidly and reached its maximum concentration within 30 min. However, SeMetO subsequently decreased and was completely reduced after 120 min (Figure 7a).

This oxidation–reduction pattern closely mirrors that observed in the direct SeMet–H_2_O_2_ reaction system, indicating that both pathways likely share common reactive intermediates. These results support the hypothesis that glucose oxidase does not directly oxidize SeMet but instead catalyzes glucose oxidation to generate hydrogen peroxide, which acts as the actual oxidizing agent responsible for SeMet conversion to SeMetO.

##### Protective Role of Catalase in SeMet Stability

The introduction of catalase exerted a pronounced protective effect on SeMet stability. Even in the presence of exogenously added hydrogen peroxide at an equimolar concentration (10 mM), no SeMetO signal was detected by LC–MS. This demonstrates that catalase effectively suppresses SeMet oxidation by rapidly decomposing H_2_O_2_ and interrupting the reactive oxygen species (ROS)-mediated oxidation cascade.

A dual-enzyme system consisting of glucose oxidase and catalase further validated this mechanism. Although H_2_O_2_ was continuously generated by glucose oxidase, its rapid removal by catalase eliminated oxidative stress and maintained a reducing microenvironment, thereby preserving SeMet integrity. These results highlight the central role of redox balance—particularly H_2_O_2_ regulation—in governing the stability of selenoamino acids.

#### 3.4.4. Implications for Selenium Stability During Food Processing

Taken together, these model-system results demonstrate that SeMet undergoes reversible oxidation to SeMetO under oxidative conditions, followed by temperature-dependent thermal reduction back to SeMet. This reversible redox mechanism explains why SeMet is retained well during bean sprout processing, even under transient oxidative stress.

More importantly, the enzymatic results indicate that enhancing reactive oxygen species (ROS) scavenging capacity, particularly catalase activity, is critical for preserving selenium speciation. From a food processing perspective, the model-system results suggest that maintaining redox homeostasis is a more effective strategy for stabilizing bioavailable selenium in enriched foods than simply reducing heat exposure.

### 3.5. Effect of Heating on SeMet Bioaccessibility in Selenium-Enriched Bean Sprouts

To clarify the effect of thermal processing on SeMet bioaccessibility, an in vitro simulated gastrointestinal digestion model was used to evaluate the impact of different heating durations (1, 3, and 5 min at 100 °C) on sprouts cultivated with Se(IV). The SeMet contents in fresh and heated samples are presented in Figure 7b.

Following simulated gastrointestinal digestion, SeMet analysis showed that heat treatment significantly enhanced SeMet bioaccessibility in both gastric and intestinal phases, with longer heating times leading to greater improvements. Samples heated for 5 min achieved approximately 71% intestinal bioaccessibility, notably higher than that of the unheated control (≈56%).

Although the efficacy of dietary selenium depends on its total content, chemical speciation, matrix interactions, and processing history—and inappropriate heating can lead to loss—optimized thermal processing, as applied here, has been shown to improve bioaccessibility. This enhancement may be attributed to heat-induced structural modifications, such as protein denaturation, cell wall disruption, and fiber softening, which could facilitate the release of protein-bound SeMet into the digestible fraction. However, this proposed mechanism requires further experimental validation.

To conclude, although short-term heating may induce transient oxidative changes in SeMet, it ultimately enhances digestive bioaccessibility by weakening matrix constraints. These findings underscore the importance of balancing selenium retention with matrix disassembly during processing to maximize the effective utilization of SeMet from selenium-enriched bean sprouts.

### 3.6. Effects of Food Matrix Components on the Thermal Reduction of SeMetO

Given the susceptibility of selenium-enriched bean sprouts to the oxidative conversion of SeMet to SeMetO, the influence of three representative matrix components—soy protein, pectin, and cellulose—on the thermal reduction of SeMetO was examined under controlled heating conditions.

The results demonstrated that all three components significantly promoted the thermal reduction of SeMetO (Figure 7c–e). At 70 °C, soy protein exhibited the strongest promoting effect (Figure 7c), followed by pectin (Figure 7e) and cellulose (Figure 7d). At 90 °C, the effectiveness ranking shifted to cellulose > protein > pectin (Figure 7c–e). At 100 °C, pectin and cellulose showed comparable promoting effects (Figure 7d,e), both exceeding that of protein (Figure 7c).

The observed temperature-dependent differences in the promoting effects of soy protein, pectin, and cellulose reflect distinct thermochemical behaviours of the individual components, as detailed below.

#### 3.6.1. Mechanistic Interpretation of Component-Dependent Effects

##### Protein

Heating denatures proteins, cleaving disulfide bonds and exposing free thiol (–SH) groups. These thiols, known for their strong reducing capacity, can directly reduce the Se=O bond in SeMetO [35]. Additionally, trace metal ions present in proteins may coordinate with thiol and carboxyl groups under heat, creating catalytic microenvironments that synergistically enhance electron transfer and accelerate SeMetO reduction.

##### Cellulose

While inert at ambient temperature, cellulose partially degrades upon heating, generating reducing carbonyl compounds (e.g., furfural, hydroxymethylfurfural) and small organic acids [36]. These products act as hydrogen or electron donors, thereby promoting the thermal reduction of SeMetO—a mechanism that explains the increasing effectiveness of cellulose at higher temperatures.

##### Pectin

Thermal treatment depolymerizes pectin into reducing sugars (e.g., galactose, arabinose) and uronic acids. D-Galacturonic acid can participate in Maillard-type reactions with amino acids, yielding reductive intermediates such as melanoidins [37]. Polysaccharide degradation fragments also possess intrinsic electron-donating ability, further facilitating selenium redox reactions. These combined effects underpin the strong promoting role of pectin in SeMetO reduction, especially at elevated temperatures.

#### 3.6.2. Collective Implications for Selenium Transformation During Thermal Processing

Collectively, these results indicate that the sprout matrix actively modulates selenium speciation during heating. By providing reductants, exposing catalytic groups, and generating reactive intermediates, matrix components enhance the thermal reduction of SeMetO to SeMet. This matrix-assisted redox regulation mechanistically explains the high retention and improved bioaccessibility of SeMet following short-term thermal processing.

This finding aligns with the established understanding that food matrix constituents—including proteins, polysaccharides, dietary fiber, metal ions, and micronutrients—play crucial roles in determining selenium stability and bioaccessibility during processing and digestion [38]. In this context, proteins may facilitate SeMet transport and release, whereas certain metals can impede selenium absorption. Polysaccharides and fiber components, on the other hand, can participate in redox reactions or alter the physicochemical microenvironment during heating.

From a processing standpoint, these insights confirm that selenium stability is not governed solely by its inherent chemistry but is significantly influenced by the surrounding food matrix. Thus, strategic control of matrix composition and heating conditions offers a viable approach to preserve—and potentially enhance—the nutritional functionality of selenium-enriched foods.

## 4. Conclusions

This study elucidates the mechanisms governing the stability of SeMet, the primary nutritional selenium species in enriched mung bean sprouts. SeMet retention is governed by two key factors: the enzymatic redox balance and food matrix interactions, with their relative importance depending on the processing condition.

Thermal processing inactivates endogenous antioxidant enzymes (e.g., CAT, PPO), driving a shift toward inorganic Se(IV). Notably, SeMet demonstrated superior thermal stability, with its retention strongly correlated with residual CAT (r = 0.975) and PPO (r = 0.884) activities, suggesting a protective role of residual enzyme activities against oxidative degradation. Conversely, storage promoted SeMet accumulation, linked to postharvest senescence processes and the enzymatic reduction of inorganic selenium.

Model studies revealed a reversible SeMet–SeMetO redox couple. SeMetO reduction followed first-order kinetics (Ea = 120.73 kJ·mol^−1^) and was accelerated by matrix components (soy protein, cellulose, pectin). Short-term heating (100 °C, 5 min) enhanced SeMet bioaccessibility from ~56% to ~71%, attributable to both SeMetO reduction and matrix-assisted release.

In conclusion, this study demonstrates that short-duration thermal processing (e.g., 100 °C for 1–3 min) can effectively modulate the enzymatic redox environment to favor the retention of organic selenium, particularly SeMet, in mung bean sprouts. Based on the observed thermostability of enzymes and selenium species, it is plausible that comparable effects could be achieved at lower temperatures (e.g., 70–90 °C) with extended treatment times. Therefore, mild thermal treatments (such as blanching at 70–90 °C for 1–5 min) are recommended as a potential strategy to preserve bioavailable selenium, though the precise time-temperature combinations require further optimization and validation in applied settings.

## Figures and Tables

**Table 1:** Pearson correlation between enzyme activity and selenium content in the Se(IV) group under thermal processing.

	Se(IV)	SeCys_2_	SeMC	SeMet	CAT	PPO
Se(IV)	1					
SeCys_2_	−0.986 **	1				
SeMC	−0.848 **	0.892 **	1			
SeMet	−0.956 **	0.983 **	0.951 **	1		
CAT	−0.979 **	0.989 **	0.866 **	0.975 **	1	
PPO	−0.769 **	0.818 **	0.971 **	0.884 **	0.794 **	1

Note: ** indicates that the correlation is significant at the 0.01 level (two-tailed).

**Table 2:** Pearson correlation between enzyme activity and selenium content in the Se(IV) group under room temperature storage (20 °C).

	Se(IV)	SeCys_2_	SeMC	SeMet	CAT	PPO
Se(IV)	1					
SeCys_2_	−0.418	1				
SeMC	−0.974 **	0.551	1			
SeMet	−0.676 *	0.876 **	0.762 *	1		
CAT	0.19	−0.967 **	−0.329	−0.783 *	1	
PPO	0.569	−0.979 **	−0.684 *	−0.944 **	0.908 **	1

Note: ** indicates that the correlation is signiffcant at the 0.01 level (two-tailed); * indicates that the correlation is signiffcant at the 0.05 level (two-tailed).

**Table 3:** Pearson correlation between enzyme activity and selenium content in the Se(IV) group under refrigerated storage (4°C).

	Se(IV)	SeCys_2_	SeMC	SeMet	CAT	PPO
Se(IV)	1					
SeCys_2_	−0.887 **	1				
SeMC	−0.760 **	0.926 **	1			
SeMet	−0.553	0.404	0.173	1		
CAT	0.939 **	−0.954 **	−0.810 **	−0.579 *	1	
PPO	0.878 **	−0.984 **	−0.892 **	−0.511	0.968 **	1

Note: ** indicates that the correlation is signiffcant at the 0.01 level (two-tailed); * indicates that the correlation is signiffcant at the 0.05 level (two-tailed).

**Figure A1:**
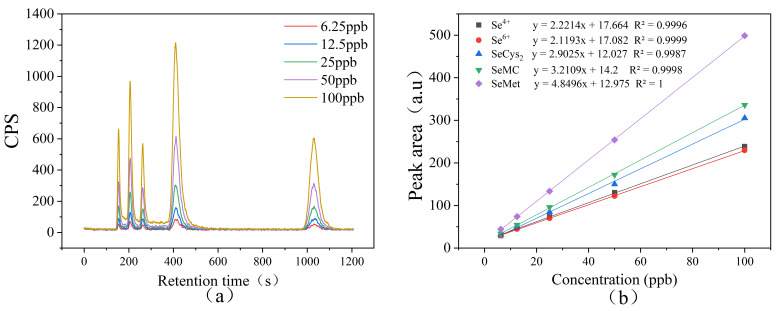
HPLC-ICP-MS chromatograms and standard curves of five selenium species standards. (**a**) Overlaid chromatograms of selenium species standards at different concentrations. (**b**) Calibration curves of selenium species standards (plot of concentration vs. response).

**Figure A2:**
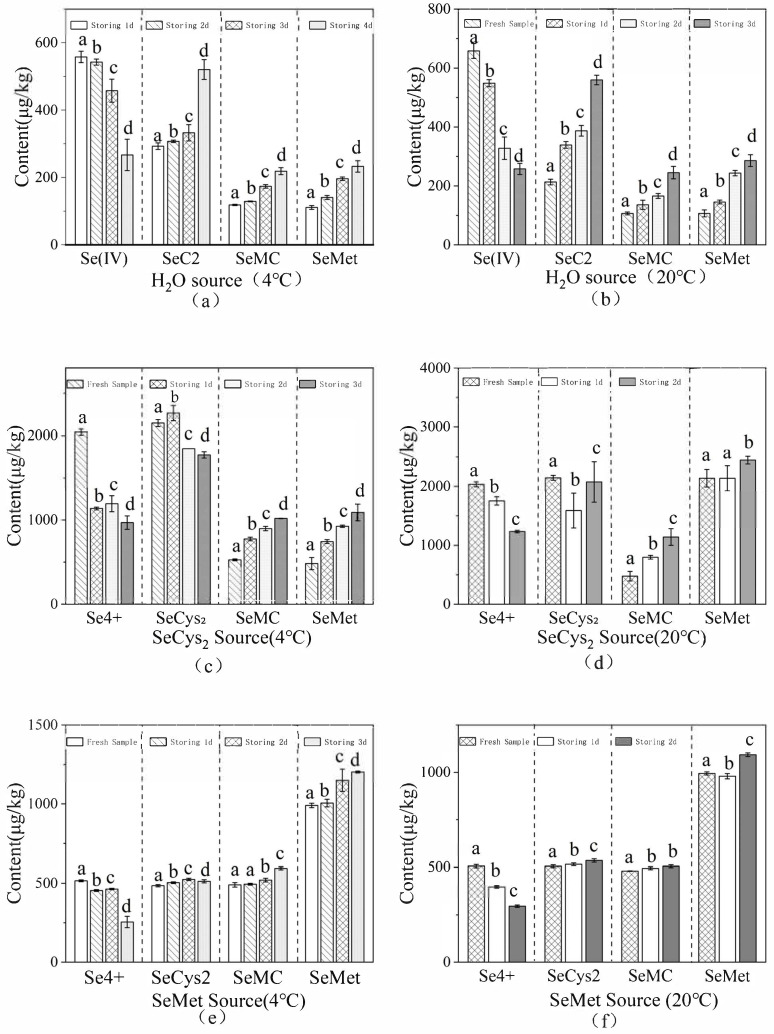
Changes in selenium speciation in bean sprouts during storage at 4 °C and 20 °C. Sprouts were cultivated with different selenium sources: (**a**,**b**) hydroponic control, (**c**,**d**) SeMet-fortified group, (**e**,**f**) SeCys_2_-fortified group. Storage temperatures: left panels (**a**,**c**,**e**) 4 °C; right panels (**b**,**d**,**f**) 20 °C. Data with different letters are significantly different according to Duncan’s test (*p* < 0.05).

## Data Availability

The original contributions presented in this study are included in the article. Further inquiries can be directed to the corresponding author.

## Appendix A

Five selenium species (SeCys_2_, SeMC, Se(IV), SeMet, and Se(VI)) were well separated using (NH_4_)_2_HPO_4_ mobile phase at pH 4.48, with retention times of 153 s, 206 s, 261 s, 411 s, and 1028 s, respectively. The peaks were clearly resolved, and the retention times were sensitive to slight variations in mobile phase pH. It is noted that values below the detection limit (LOD: 140–220 μg kg^−1^) are shown for trend reference only and were excluded from statistical analysis.

## Appendix B

Changes in selenium speciation in bean sprouts during storage at 4 °C and 20 °C. This Figure compares the sprouts cultivated with different selenium sources: the hydroponic control group, the SeMet-fortified group, and the SeCys_2_-fortified group.

## Appendix C

Changes in the activities of PPO and CAT in bean sprouts during storage at 4 °C and 20 °C. This Figure compares the enzymatic profiles of sprouts cultivated with two different selenium sources: the SeMet-fortified group and the SeCys_2_-fortified group.
